# Bilateral Pneumonia

**Published:** 1895-05

**Authors:** Otto Noack

**Affiliations:** Reading, Pa.


### REPORTS OF CASES.

### BILATERAL PNEUMONIA—DEATH.

BY OTTO NOACK, READING, PA.

The subject was a gray horse suffering with pneumonia bilateralis. Received him for treatment after transportation. The horse hung his head and had a stupid appearance, mouth dry and hot, temperature 105.8° F., breathing with difficulty and very rapid. By auscultation of the lungs no sound to be heard in the middle parts. Pulse quick and feeble. The animal staggered to keep from falling. These symptoms showed that death was certain and close. Died the following morning.

*Dissection.* Animal in poor condition. In the abdominal cavity no abnormal contents could be found. The viscera were in normal position and state. The tissues of the stomach were swollen in the pyloric portion, and showed red spots. The spleen was somewhat enlarged and soft in consistence. Pulp soft and hard, a brownish red color. The liver was larger than normal and rims curled, and the organ appeared hardened, deep red and dull in appearance. Centre of the acini were brownish red, the peripheral part a grayish color. In different parts the commencing and ending of the acini could not be distinguished. The kidneys were enlarged, brittle to the touch, dry and dull looking. The glomeruli could be easily recognized in the cortical substances as very small red points. No abnormal substance could be found in the serosa of the thoracic cavity. The heart muscle was dry and brittle, grayish red and dull, a condition also recognized in the gluteal muscles. Both lungs on the lower parts of the middle third were non-elastic and tough. By pressing on the cut surface a grayish white fluid escaped. The other parts of both lungs were normal on the surface and on section. In the lower part of the trachea traces of the above-mentioned fluid could be seen.

*Anatomical Diagnosis.* Pneumonia catarrhalis bilateralis; œdema pulmonum myocarditis et myositis parenchymatosa; gastritis hæmorrhagica; intumescentia lienis; hepatitis et nephritis parenchymatosa.

